# Time Trend and Demographic and Geographic Disparities in Childhood Obesity Prevalence in China—Evidence from Twenty Years of Longitudinal Data

**DOI:** 10.3390/ijerph14040369

**Published:** 2017-03-31

**Authors:** Peng Jia, Hong Xue, Ji Zhang, Youfa Wang

**Affiliations:** 1 Department of Earth Observation Science, Faculty of Geo-Information Science and Earth Observation (ITC), University of Twente, Enschede 7500, The Netherlands; jiapengff@hotmail.com; 2 Fisher Institute of Health and Well-Being, Systems-Oriented Global Childhood Obesity Intervention Program, College of Health, Ball State University, Muncie, IN 47306, USA; hxue@bsu.edu; 3 National Institute for Nutrition and Health, Chinese Center for Disease Control and Prevention, Beijing 100000, China; shion_zhang@163.com; 4 Department of Nutrition and Health Sciences, College of Health, Ball State University, Muncie, IN 47306, USA

**Keywords:** China, child, obesity, overweight

## Abstract

Childhood overweight and obesity (ow/ob) has become a serious threat to many countries, including China. However, limited evidence was obtained from longitudinal data in China. This study examined the secular trends and geographic variation in the prevalence of ow/ob and obesity only, and age, gender, and urban-rural disparities among school-aged children across China. Data from children aged 6–17 surveyed in China Health and Nutrition Survey (CHNS) from 1991 (n = 2712) to 2011 (n = 1054) were used. Overweight and obesity were defined based on the International Obesity Task Force (IOTF) recommended Asian age-sex-specific BMI cut-off-points. We found that: (1) childhood ow/ob and obesity prevalence increased from 11.7% to 25.2% and from 2.8% to 10.1% during 1991–2011, respectively; (2) children aged 6–12 experienced a 1.3 and 1.6 times increase in ow/ob and obesity prevalence than children aged 13–17, respectively; (3) the urban-rural gap in ow/ob prevalence widened; (4) ow/ob prevalence in boys was higher and increased faster than in girls, especially in an urban setting; and (5) geographic variation was observed with faster increases in more economically developed east, central and northeast regions than in the less developed west. The findings added more nuances to the picture of temporal changes in ow/ob prevalence among Chinese children.

## 1. Introduction

Childhood overweight and obesity (ow/ob) has become a serious threat to global health. During 1980–2013, the combined prevalence of childhood ow/ob has increased from 16.9% to 23.8% for boys and from 16.2% to 22.6% for girls in developed countries. It also increased dramatically in developing countries from 8.1% to 12.9% for boys and from 8.4% to 13.4% for girls [1]. Childhood obesity has many short- and long-term health consequences, and it has been recommended that efforts should be more focused on children than on adults for obesity prevention [2,3,4,5,6,7,8].

With the rapid economic development since the 1980s, ow/ob rates have been increasing in China. Some previous research examined the temporal changes in ow/ob prevalence, and age, gender, and urban-rural disparities among Chinese school-aged children [8,9,10]. For example, from 1985 to 2000, the combined prevalence of ow/ob in school-aged children increased from 1.6% to 23.6% for boys and from 1.8% to 13.6% for girls in coastal big cities, and increased from 0.6% to 12.4% for boys and from 1.2% to 7.2% for girls in inland big cities [11]. A meta-analysis shows that the prevalence of childhood ow (ob) increased from 1.8% (0.4%) in 1981–1985 to 13.1% (7.5%) in 2006–2010 in China [12]. However, limited evidence was obtained from community-based longitudinal data.

Using data from the longitudinal China Health and Nutrition Survey (CHNS) conducted from 1991 to 2011, this study examined the time trends and geographic variation in the prevalence of ow/ob and obesity only, and the age, gender, and urban-rural disparities among school-aged children across China. Our findings added more nuances to the picture of temporal changes in ow/ob prevalence among Chinese school-aged children.

## 2. Materials and Methods

### 2.1. Study Design and Study Sample

**Figure 1:**
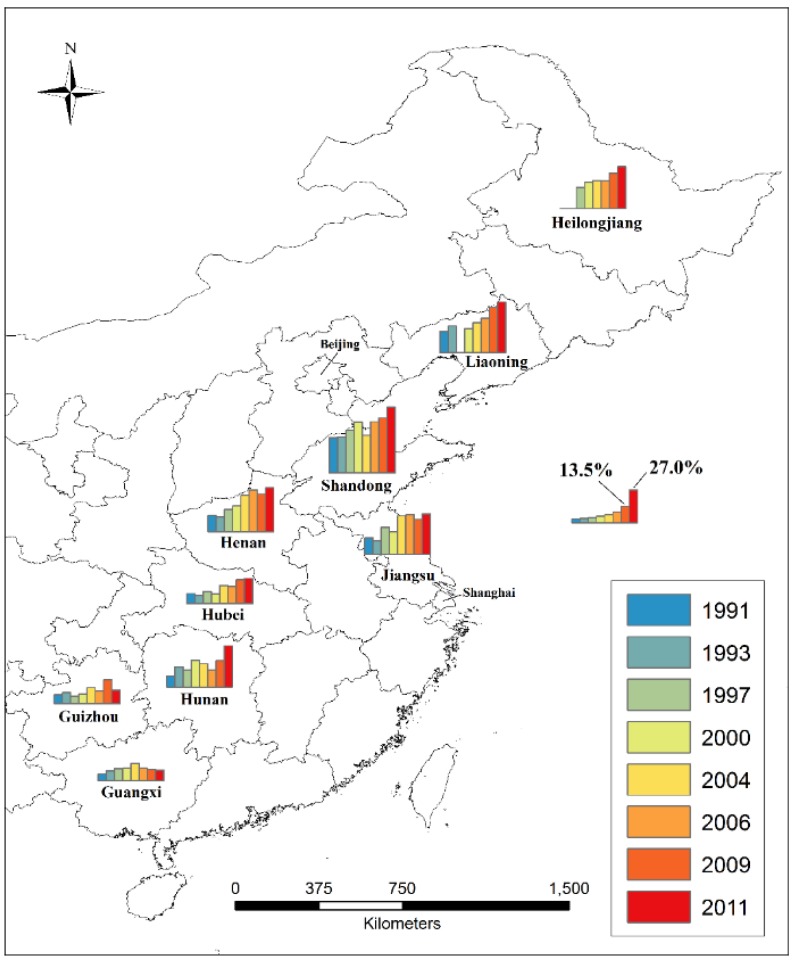
Nine Chinese provinces in which China Health and Nutrition Survey (CHNS) was conducted, with the combined prevalence (%) of overweight and obesity calculated for each survey year among children aged 6–17 sampled in each province, where overweight and obesity were defined based on the 2012 IOTF recommended age-sex-specific cut-offs corresponding to BMI ≥ 23 and ≥ 27 at age 18, respectively [14].

The CHNS is an ongoing open cohort study initiated in 1989, and was designed to examine the population’s health and nutritional status in eight provinces across China (Figure 1), which varied substantially in such factors as geography and economic development, including Liaoning (not surveyed in 1997), Shandong, Henan, Jiangsu, Hubei, Hunan, Guangxi, and Guizhou [13]. Heilongjiang was enrolled as a ninth province in 1997 (Figure 1).

In each province, cities and counties were stratified by income (low, middle, and high) and two cities and four counties were selected by a weighted sampling scheme [13]. Four communities from each city/county were selected randomly (urban/suburban neighborhoods in cities and villages/townships in counties); in each community, 20 households were randomly selected and followed up in eight subsequent waves from 1991–2011 (Table 1). In some cases, new households were recruited to substitute for those who migrated out of the community for different reasons.

Records of children aged 6–17 in each wave were extracted for this study. The numbers of the children extracted were 2712 (1991), 2580 (1993), 2495 (1997), 2359 (2000), 1544 (2004), 1220 (2006), 1113 (2009), and 1054 (2011). A total of 4507 (60.4%) of 7460 different children were followed through at least two consecutive waves, and 43.6% of which (1966 out of 4507) were followed up for three or more consecutive waves, respectively.

Children’s weight and height were measured by at least two trained health professionals who followed standard protocol and techniques, with one taking the measurements and another recording the readings [15]. Weight was measured in light indoor clothing without shoes to the nearest tenth of a kilogram with a beam balance scale, and height was measured without shoes to the nearest tenth of a centimeter with a portable stadiometer.

### 2.2. Key Study Variables

Overweight and obesity were defined based on the International Obesity Task Force (IOTF) recommended Asian age-sex-specific cut-offs corresponding to BMI ≥ 23 and 27 at age 18, respectively [14]. Overall children were stratified by age (≤12 and >12), gender (boy and girl), urbanicity (urban and rural), and region (northeast, east, central, and west [16]). Ow/ob and obesity prevalence were calculated for each category in each wave.

### 2.3. Statistical Analysis

A linear regression model was fit to assess over-time changes in prevalence and the average annual increase among the overall sample during 1991–2011, with adjusted R^2^ calculated for assessing the performance of the model. Separate linear regression models were also fit by age group, gender, urbanicity, and region, to examine potential differences in prevalence and average annual increase across these characteristics. Additionally, gender differences were further explored within each urbanicity category; in other words, urbanicity differences were tested within each gender. To demonstrate disparities, prevalence ratios in 1991 and 2011, and annual increase ratios between age groups, genders, urbanicity categories, and regions were calculated.

## 3. Results

### 3.1. Overall Trend

**Figure 2:**
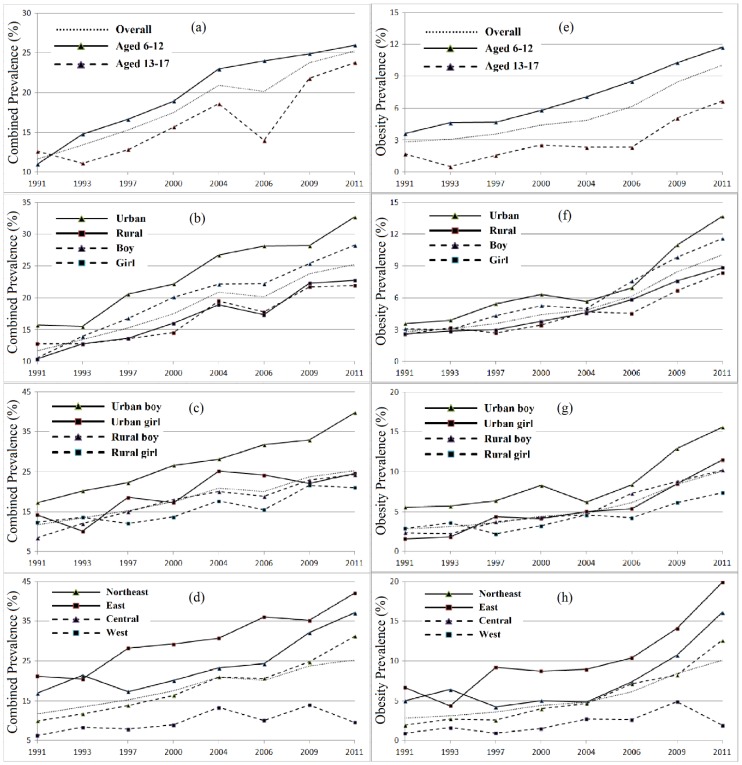
Time trend (1991 to 2011) and disparities across population groups in combined prevalence of overweight and obesity (ow/ob) and prevalence of obesity among children aged 6–17 in China, where overweight and obesity were defined based on the 2012 IOTF recommended age-sex-specific cut-offs corresponding to BMI ≥ 23 and ≥ 27 at age 18, respectively [14]: (**a**) ow/ob prevalence by age group; (**b**) ow/ob prevalence by gender and urbanicity, separately; (**c**) ow/ob prevalence by gender and urbanicity; (**d**) ow/ob prevalence by region; (**e**) obesity prevalence by age group; (**f**) obesity prevalence by gender and urbanicity, separately; (**g**) obesity prevalence by gender and urbanicity; (**h**) obesity prevalence by region.

The prevalence of ow/ob along all children aged 6–17 more than doubled from 11.7% in 1991 to 25.2% in 2011 (Figure 2a), with an average annual increase of 0.7% (Table 1). The prevalence of obesity more than tripled from 2.8% in 1991 to 10.1% in 2011 (Figure 2e), with an average annual increase of 0.3% (Table 1).

### 3.2. Age Disparity

Children aged 6–12 experienced a faster annual increase than those aged 13–17 in both ow/ob (2.2% versus 1.6%) and obesity prevalence (1.2% versus 0.7%) over the 20-year period (Figure 2a,e). Despite slight differences in ow/ob prevalence, obesity prevalence of those aged 6–12 was on average about 2–4 times higher than those aged 13–17 (an abnormally large difference in 1993 was due to the small number of children aged 13–17 in that wave) (Table 1). The obesity prevalence ratio for children aged 6–12 versus 13–17, however, generally shrunk over time.

### 3.3. Gender Disparity

Ow/ob prevalence increased from 10.6% to 28.3% in boys, and from 12.8% to 21.9% in girls during 1991–2011 (Figure 2b and Table 1); obesity prevalence more than tripled in boys, from 3.1% to 11.6%, and in girls, from 2.6% to 8.4% (Figure 2f and Table 1). Prevalence in boys also increased at a faster annual rate than in girls (0.8% versus 0.5% for ow/ob; 0.4% versus 0.3% for obesity). Ow/ob and obesity prevalence ratios for boys versus girls had been constantly >1.0 since 1993 and 1997, respectively (Table 1).

Different growth trends between genders were observed in urban and rural settings (Figure 2c,g). In urban regions, boys had higher ow/ob prevalence (1.1–2.0 times) and an apparently faster annual growth rate (1.0% versus 0.6%) than girls; obesity prevalence in boys was also consistently higher than in girls (1.2–3.5 times), but with a similar growth rate of 0.4% (Table 1). In rural regions, both ow/ob and obesity prevalence ratios for boys versus girls had reversed since 1997 (0.89 in 1993 versus 1.24 in 1997 for ow/ob; 0.61 in 1993 versus 1.69 in 1997 for obesity), which might account for the between-gender reversal in 1997 among all children; hence, obesity prevalence in boys increased at a faster average annual rate than in girls (0.7% versus 0.5% for ow/ob; 0.4% versus 0.2% for obesity).

### 3.4. Urban-Rural Disparity

During 1991–2011, ow/ob prevalence more than doubled in urban children, from 15.7% to 32.7%, and in rural children, from 10.4% to 22.8% (Figure 2b and Table 1); obesity prevalence more than tripled in urban children, from 3.6% to 13.7%, and in rural children, from 2.6% to 8.8% (Figure 2f and Table 1). The urban-rural gap widened in both ow/ob (15.7% versus 10.4% in 1991; 32.7% versus 22.8% in 2011) and obesity prevalence (3.6% versus 2.6% in 1991; 13.7% versus 8.8% in 2011).

Urban boys had higher ow/ob prevalence (1.4–2.0 times) and a faster average annual growth rate (1.0% versus 0.7%) than rural boys, and urban girls also had higher ow/ob prevalence (0.7–1.6 times) and a slightly faster growth rate (0.6% versus 0.5%) than rural girls (Figure 2c,g). Similarly, urban boys had higher obesity prevalence (1.2–2.6 times) than rural boys, but with a similar growth rate of 0.4%. The obesity prevalence ratio for urban girls versus rural girls had reversed since 1997 (0.54 in 1991 versus 1.56 in 2011), hence urban girls had a faster growth rate (0.4% versus 0.2%) than rural girls (Table 1).

### 3.5. Geographic Disparity

The east region saw the highest ow/ob (21.2%) and obesity prevalence (6.7%) in 1991, followed by the northeast (16.9% for ow/ob and 5.0% for obesity), central (9.9% and 1.9%), and west regions (6.3% and 0.9%) (Figure 2d,h). Ow/ob prevalence in eastern (including northeast and east) regions was 2–3 times higher than in central and west regions, and obesity prevalence in eastern regions was 3–5 times higher than in central and west regions (Table 1). The west region also saw an apparently slower average annual increase than other regions in ow/ob (0.3% versus 0.8%–1.0%) and obesity prevalence (0.1% versus 0.4%–0.6%), which further widened the existing regional gap in 1991, and led to dramatically lower ow/ob and obesity prevalence in western regions than in other regions in 2011 (9.6% versus 31.2%–42.0% for ow/ob; 4.9% versus 8.3%–14.1% for obesity) (Table 1).

## 4. Discussion

This study found that ow/ob and obesity prevalence in Chinese children have been increasing over time, and there are age, gender, urban-rural, and regional disparities in the prevalence and increasing rates. Age differences identified in this study were in line with previous findings [17,18]. Younger children tended to be less active, especially during weekends, which may partly explain their higher obesity prevalence [19]. The ow/ob prevalence in boys was higher and increased faster than in girls, especially in the urban settings. Boys in China have been described as being particularly vulnerable to obesity because of genetic, behavioral, and socio-cultural reasons [10,20]. However, the gender ratios in general and in the urban setting found in our study were on average lower than ratios found by previous studies [8,10]. Geographic variation was observed with faster increases in east, central and northeast regions than in the less economically developed west region. Eastern China witnessed higher prevalence and faster increases, which may be due to both economic reasons (eastern China is the most developed region among the four economic regions [21]) and energy balance related behaviors (northeastern people tended to stay indoors and consume high-energy food during winter time due to cold temperature [22]).

Urban-rural disparities were also evident and became larger over time, but not as large as the ones found from other cross-sectional studies, where each province as the basic unit was designated as either urban or rural [9]. Our study classified urban/rural residence at the neighborhood level based on the location of children’s residence, which allows us to reveal more accurate changes in urban-rural ratios. The initiation of a series of policies for liberalizing pricing and food procurement by Chinese government since the end of the 1980s and beginning of the 1990s has ignited an increasingly abundant food supply in urban areas, such as abolition of the urban grain rationing system in 1993 [23]. Although urban retail grain price subsequently soared and matched the retail price on rural free markets, a dramatic increase in annual per capita disposable income of both urban and rural households elevated the affordability during 1990–2011, which was experienced in parallel with the increasingly widened urban-rural gap in ow/ob and obesity prevalence. For example, despite an increase in price per ton of rice in urban regions from 989 CNY (Yuan, Chinese monetary unit) from 1980–1993 to 2144 CNY from 1994–2001, with only a slight change in rural regions from 2069 to 2145 CNY, the annual per capita disposable income of urban and rural households also increased accordingly, from 1510 to 21,810 CNY and from 686 to 6977 CNY, respectively [23]. According to the official historical average exchange rates, one CNY was approximately 0.19, 0.12, and 0.15 US dollars in 1991, 2001, and 2011, respectively [24].

There are some limitations in this study. A major one is that CHNS covers only nine instead of all 35 provinces/municipalities, and thus we could not fully examine geographic variations in China. Also, because we only focused on the children’s part (aged 6–17) of the CHNS dataset, some children were unavoidably excluded from our dataset due to going above the age of 17. Most of these also left the cohort due to attending colleges or left home for reasons such as starting working [13]. More details of attrition of the CHNS cohort can be found in the cohort profile [13]. As the loss to follow up during the 20 years follow-up period was largely random, our results may not be much affected. Nevertheless, we cannot completely rule out that the BMIs of children who stayed in the cohort differed from of those who dropped out. However, CHNS data collection focused on the same communities over the study period, which reduced the influences of socio-economic and, in particular, environmental disparities on individuals (between-individual variation) [25]. Thus, under the community-based study design adopted by CHNS, it was reasonable to assume that the new households recruited to substitute for those who migrated out of the community were comparable with the migrated ones. Also, urban and rural neighborhoods were clearly defined and differentiated in CHNS, which was rare in other study designs and made it possible to provide a better view of urban-rural gaps in ow/ob and obesity prevalence.

## 5. Conclusions

The prevalence of childhood ow/ob and obesity only have increased in China. Moreover, there are different increasing trends across populations and regions in China (i.e., a faster growth in boys, the younger, urban children, and eastern and central regions). This study comprehensively examined school-aged children based on the CHNS data, and provided more nuances to the picture of temporal changes in ow/ob prevalence among Chinese school-aged children. This provided new, useful insight into the problems, and highlighted the related disparities and the needs of effective intervention programs in China, in fighting the growing obesity epidemic. Future interventions should be tailored to different subpopulations based on distinct increases in obesity prevalence, to more efficiently control the childhood obesity epidemic and reduce the disparities in China.

## Figures and Tables

**Table 1:** Changes over time (1991 to 2011) in combined prevalence of overweight and obesity ^a^ and prevalence of obesity among children aged 6–17 in China.

	Prevalence (%)								Annual Increase		Prevalence Ratio ^†^		
	1991	1993	1997	2000	2004	2006	2009	2011	S ^††^	R^2^	1991	2011	S ^†††^
A. Combined prevalence of overweight and obesity													
All ^b^	11.7	13.4	15.3	17.5	20.9	20.1	23.8	25.2	0.7 *	0.98			
Age													
6–12	11.0	14.8	16.6	18.9	23.0	24.0	24.9	26.0	2.2 *	0.96	0.87	1.09	1.32
13–17	12.6	11.1	12.8	15.6	18.6	14.0	21.8	23.8	1.6 *	0.76	ref	ref	ref
Gender													
Boy	10.6	14.0	16.8	20.1	22.1	22.2	25.4	28.3	0.8 *	0.97	0.82	1.29	1.57
Girl	12.8	12.8	13.6	14.5	19.5	17.8	21.7	21.9	0.5 *	0.89	ref	ref	ref
Urbanicity													
Urban	15.7	15.5	20.6	22.2	26.7	28.2	28.2	32.7	0.8 *	0.97	1.51	1.44	1.42
Rural	10.4	12.8	13.6	16.0	18.9	17.3	22.3	22.8	0.6 *	0.94	ref	ref	ref
Urban													
Urban boy	17.2	20.3	22.3	26.6	28.1	31.8	32.9	39.7	1.0 *	0.95	1.21	1.62	1.51
Urban girl	14.2	10.1	18.6	17.2	25.1	24.2	22.0	24.6	0.6 *	0.73	ref	ref	ref
Rural													
Rural boy	8.5	12.1	15.0	18.1	20.1	18.9	22.9	24.3	0.7 *	0.94	0.69	1.16	1.56
Rural girl	12.4	13.6	12.1	13.7	17.7	15.5	21.6	21.1	0.5 *	0.75	ref	ref	ref
Region ^††††^													
Northeast	16.9	21.4	17.3	20.1	23.2	24.3	32.1	37.1	0.8 *	0.71	2.69	3.87	3.23
East	21.2	20.4	28.3	29.3	30.7	36.1	35.2	42.0	1.0 *	0.92	3.36	4.39	3.72
Central	9.9	11.7	13.9	16.3	20.9	20.6	24.8	31.2	0.9 *	0.93	1.58	3.26	3.62
West	6.3	8.3	7.9	8.9	13.3	10.1	13.9	9.6	0.3 *	0.45	ref	ref	ref
B. Obesity prevalence													
All ^b^	2.8	3.1	3.6	4.4	4.9	6.1	8.4	10.1	0.3 *	0.84			
Age													
6–12	3.6	4.6	4.7	5.8	7.1	8.5	10.3	11.7	1.2	0.96	2.09	1.76	1.65
13–17	1.7	0.5	1.6	2.6	2.3	2.3	5.1	6.7	0.7	0.74	ref	ref	ref
Gender													
Boy	3.1	3.0	4.3	5.3	5.0	7.6	9.8	11.6	0.4 *	0.85	1.20	1.39	1.59
Girl	2.6	3.2	2.7	3.4	4.7	4.5	6.7	8.4	0.3 *	0.77	ref	ref	ref
Urbanicity													
Urban	3.6	3.9	5.4	6.3	5.7	7.0	11.0	13.7	0.4 *	0.75	1.37	1.55	1.42
Rural	2.6	2.9	3.0	3.8	4.6	5.8	7.6	8.8	0.3 *	0.86	ref	ref	ref
Urban													
Urban boy	5.5	5.7	6.4	8.3	6.2	8.4	12.9	15.6	0.4 *	0.65	3.51	1.36	1.01
Urban girl	1.6	1.8	4.4	4.1	5.0	5.4	8.5	11.5	0.4 *	0.82	ref	ref	ref
Rural													
Rural boy	2.3	2.2	3.7	4.3	4.6	7.3	8.8	10.2	0.4 *	0.89	0.81	1.39	1.95
Rural girl	2.9	3.6	2.2	3.2	4.6	4.2	6.1	7.4	0.2 *	0.67	ref	ref	ref
Region ^†††^													
Northeast	5.0	6.4	4.2	5.0	4.8	7.3	10.7	16.1	0.4 *	0.45	5.47	8.40	3.32
East	6.7	4.4	9.2	8.8	8.9	10.4	14.1	19.9	0.6 *	0.70	7.31	10.40	4.63
Central	1.9	2.7	2.6	4.0	4.7	7.1	8.3	12.6	0.5 *	0.79	2.11	6.59	3.72
West	0.9	1.6	0.9	1.5	2.7	2.6	4.9	1.9	0.1	0.38	ref	ref	ref

Note: ^a^ Overweight and obesity were defined based on the 2012 IOTF recommended age-sex-specific cut-offs corresponding to BMI ≥ 23 and ≥ 27 at age 18, respectively [14]; ^b^ Numbers of children extracted from the China Health and Nutrition Survey (CHNS) were 2712 for 1991; 2580 for 1993; 2495 for 1997; 2359 for 2000; 1544 for 2004; 1220 for 2006; 1113 for 2009; and 1054 for 2011; * Significant at *p* < 0.05; ^†^ Ratio of prevalence in 1991/2011 to the reference group (ref); ^††^ Average annual increase during 1991–2011; ^†††^ Ratio of average annual increase to the reference group (ref); ^††††^ Northeast (Heilongjiang, Liaoning), East (Shandong, Jiangsu), Central (Henan, Hubei, Hunan), and West of China (Guizhou, Guangxi).
